# The complete and annotated mitochondrial genome of *Hemileia vastatrix* Race I, causal agent of coffee leaf rust

**DOI:** 10.12688/f1000research.177569.2

**Published:** 2026-07-15

**Authors:** Gustavo A. Marín-Ramírez, Carlos E. Maldonado, Beatriz E. Padilla Hurtado, Sergio H. Brommonschenkel

**Affiliations:** 1 Plant Pathology /Laboratory of Genetics and Genomics of Plant-Pathogen Interaction, BIOAGRO, Viçosa, State of Minas Gerais, 36570-900, Brazil; 2 Plant Pathology, National Centre of Coffee Research, Chinchiná, Caldas, 170009, Colombia; 3 Catholic University of Manizales, Manizales, Caldas, 170003, Colombia

**Keywords:** Hemileia vastatrix, Mitochondrial genome, Coffee leaf rust, PacBio HiFi, Fungicide resistance, Cytochrome b, atp8, Genome assembly

## Abstract

*Hemileia vastatrix* is the fungal pathogen responsible for coffee leaf rust (CLR), the most economically important disease of *Coffea arabica* worldwide. Recently, the nuclear genome of this fungus was completely deciphered. However, the mitochondrial genome of *H. vastatrix* has remained undercharacterized. Here, we present the complete, circularized mitochondrial genome of *H. vastatrix* Race I (isolate HvRI), assembled using a hybrid approach combining PacBio HiFi long reads and BGIseq short reads. The genome is 173,525 bp in length with a GC content of 33.1% and encodes 41 functional genes, including 15 protein-coding genes, 2 rRNAs, and 24 tRNAs. The assembly reveals significant structural complexity, driven by intron expansion in the *cox1* and *cob* genes. Notably, the *atp8* gene contains a group II intron, rare for this locus, whose internal open reading frame displays evidence of pseudogenization via internal stop codons.. We also characterized a putative replication initiation zone (~1.2 kb) defined by a poly-G homopolymer and conserved regulatory motifs. The mitogenome of the HvRI isolate does not contain *cob* mutations that lead to amino acid substitutions G143A and F129L associated with the quinone outside inhibitor (QoI) fungicide resistance. This high-quality mitogenome is an important resource for comparative mitogenomics, population diversity studies, and the molecular surveillance of QoI fungicide resistance.

## Introduction

*Hemileia vastatrix* Berk. and Br., causal agent of coffee leaf rust (CLR), is the most devastating pathogen in global coffee production. ^1^ Although recent long-read sequencing efforts have resolved its large and repeat-rich nuclear genome (~748 Mb), ^2^ the mitochondrial genome of *H. vastatrix* remains undercharacterized.

Mitochondria are double-membrane organelles, present in high copy numbers per cell. They are essential for fungal metabolism and the site for oxidative phosphorylation. ^3^ In sexual eukaryotes, mitochondrial inheritance is predominantly uniparental; however, in fungi, both uniparental and biparental inheritance modes have been reported. ^4^ Another distinction across the tree of life is the mutation rate: while mitochondrial genomes evolve faster than nuclear genomes in animals, the opposite has been reported in specific fungal species. ^5^ Predominantly asexual species of the division Basidiomycota present SNP frequencies up to 7.69% in nuclear genomes but only up to 4.41% in their mitochondrial counterparts. ^5^ This slower mutation rate makes mitochondrial DNA markers useful for resolving deep evolutionary relationships between species. ^6– 8^ Despite the slow mutation rate in Basidiomycota, high variability in genome size, gene order, and intron content has been found while maintaining a conserved core set of protein-coding genes, this genomic plasticity is valuable not only for phylogenetics of distant species but also for population genomics within a species. ^9^

In fungal phytopathogens, mitochondria are involved in virulence through the regulation of growth and resistance to antimicrobial compounds. ^6, 9^ Strobilurin fungicides, also known as Quinone outside Inhibitors (QoI), suppress mitochondrial respiration by binding at the Qo site of cytochrome b (cob) within the cytochrome bc _1_ complex (cyt bc _1_), located in the inner mitochondrial membrane. This binding blocks electron transfer between cob and cytochrome c _1_, which disrupts ATP production. In fungi, the cyt bc _1_ comprises 10 or 11 subunits with a combined molecular mass of approximately 240 kD; all subunits are nuclear-encoded except *cob*, which is encoded by the mitochondrial genome. ^10, 11^ Single-point mutations within the *cob* gene confer strobilurin resistance. The most significant mutation leads to the amino acid substitution of glycine (G) to alanine (A) at position 143 (G143A) of cob and confers a high level of resistance. ^10, 11^ Additional mutations, such as F129L and G137R, also reduce the efficacy of chemical control based on QoI fungicides. ^12, 13^

Although a mitochondrial sequence was reported in a contig-level genome assembly of the *H. vastatrix* isolate Hv178a, ^14^ no complete, annotated, and curated mitochondrial genome has been made publicly available. In the *H. vastatrix* Race I genome, ^2^ the mitogenome was not assembled into a single sequence, requiring additional analysis. Here, we present a complete and circularized mitochondrial genome of *H. vastatrix* Race I, expanding the genomic resources for this fungus and providing a reference for comparative genomics and applied CLR management.

## Methods

**Biological material.** A single isolate of *H. vastatrix*, identified as Race I (HvRI), was obtained from symptomatic leaves in a *Coffea arabica* var. Caturra plantation in the rural area of Pereira (Risaralda, Colombia) at an altitude of approximately 1,800 m a.s.l. (4°44′46.25″N, 75°36′14.59″W). The pathogen was multiplied on susceptible coffee plants (var. Caturra) maintained at 23 °C and 80% relative humidity to promote sporulation. Fresh urediniospores were harvested, vacuum-dried, and stored at −80 °C. Further details on the sample are presented in Angel *et al.* (2023) ^2^ and in the NCBI BioSample record SAMN32232808.

**DNA isolation and sequencing.** High molecular weight genomic DNA isolation, library construction, and sequencing were performed by the Arizona Genomics Institute (AGI, University of Arizona, USA). DNA was isolated from 3 g of urediniospores using a modified CTAB protocol. ^15^ A PacBio SMRTbell library was prepared for HiFi sequencing and run on a Sequel II system using two Single-Molecule Real-Time (SMRT) cells, targeting an expected 70X coverage of the nuclear genome. Short-read sequencing was also performed for HvRI. DNA was isolated using the DNeasy Plant Kit (Qiagen), and 50 million 150 bp paired-end (PE) reads were generated using the BGIseq/DNBseq platform by Complete Genomics (California, USA).

**Mitogenome assembly.** The mitochondrial genome was assembled using a hybrid approach. Initially, raw BGIseq reads (SRA SRR22911637) were adapter-trimmed using Trimmomatic v0.40. ^16^ A *de novo* assembly was then performed using NOVOPlasty v4.3.516, ^17^ which utilizes internal read quality filtering, using the *cob* gene sequence of *Puccinia striiformis* f. sp. *tritici* isolate CYR32 (GenBank MH891489.1) as a seed. The resulting contig was annotated with MFannot v2.017 ^18^ using Genetic Code 4 (Mold, Protozoan, and Coelenterate Mitochondrial). The NADH dehydrogenase subunit 4 ( *nad4*) gene from this preliminary assembly was subsequently used as a seed for a second round of NOVOPlasty. Finally, prior to error correction, the raw reads underwent quality trimming with Trimmomatic (leading/trailing Q < 20; 4-bp sliding window average Q ≥ 30; minimum length 100 bp). These high-quality reads were mapped to the draft assembly using BWA-MEM v0.7.17, ^19^ and the assembly was polished with Pilon v1.24. ^20^

The resulting short-read assembly served as a reference to recruit PacBio HiFi reads (SRA SRR22911636) using minimap2. ^21, 22^ Reads with a mapping quality of at least Q30 were extracted by Samtools v1.21 ^23^ and *de novo* assembled using NextDenovo v2.5.5 ^24^ and NOVOLoci v0.5. ^17^ Although NOVOLoci is optimized for Oxford Nanopore (ONT) data, its seed-and-extend algorithm was experimentally applied here to the PacBio HiFi reads.

Contigs obtained from NextDenovo and NOVOLoci were aligned to the NOVOPlasty assembly to verify mitochondrial identity. The selected sequences were self-aligned using the nucmer module of MUMmer v3.23, ^25^ and coordinates extracted with the show-coordinates utility were used to trim overlaps and define the circular genome. The three independent assemblies were aligned using MAFFT v7.525 ^26^ and manually inspected using Unipro UGENE ^27^ to resolve discrepancies. The final consensus sequence, derived primarily from the NextDenovo assembly, was selected for annotation.

**Functional annotation.** The mitochondrial genome was annotated using MFannot v2.0 (Genetic Code 4) ^18^ and MITOS v2.1.10 (Genetic Code 4; RefSeq 89 Fungi). ^28, 29^ Predicted features from both pipelines, including protein-coding genes (PCGs), transfer RNAs (tRNAs), and ribosomal RNAs (rRNAs), were merged and manually curated. Coding sequences (CDSs) were inspected for start and stop codon integrity. To verify gene boundaries, sequences were aligned using BLASTX ^30^ against the NCBI nr database. In cases of potential truncations or missing regions, particularly regarding fungal orthologs in the order Pucciniales, open reading frames (ORFs) were manually inspected in all three relevant reading frames, and intron-exon boundaries were refined through comparative analysis.

## Results

**Mitochondrial genome assembly and validation**. The *de novo* assembly using BGIseq short reads and NOVOPlasty produced a single circular contig of 173,497 bp with an average coverage of 2,411X (2,769,734 aligned reads). A second assembly using a conspecific seed ( *nad4* derived from the first round) yielded an identical length (173,497 bp) and consistent coverage depth (2,392X), confirming the stability of the short-read assembly graph.

Polishing of the BGIseq assembly with Pilon identified and corrected three single nucleotide polymorphisms (SNPs), validating 99.87% (173,266/173,497 bases) of the initial sequence as error-free. To further verify structural integrity and sequence accuracy, an independent assembly was generated using PacBio HiFi reads. The HiFi-based assembly, generated by NextDenovo and NOVOLoci, produced a circularized sequence of 173,525 bp with 1,240X coverage.

Comparative alignment between the polished short-read assembly and the HiFi assembly revealed a high degree of concordance. The minor length discrepancy (28 bp difference) was attributed to small indels (1–2 bp) located exclusively in homopolymeric regions, which are known artifacts in short-read assemblies. Crucially, the three SNPs corrected by Pilon in the short-read assembly were confirmed by the HiFi data. Due to the superior handling of repetitive regions by long-read sequencing, the HiFi consensus (173,525 bp) was selected as the final reference sequence.

**Figure 1.**
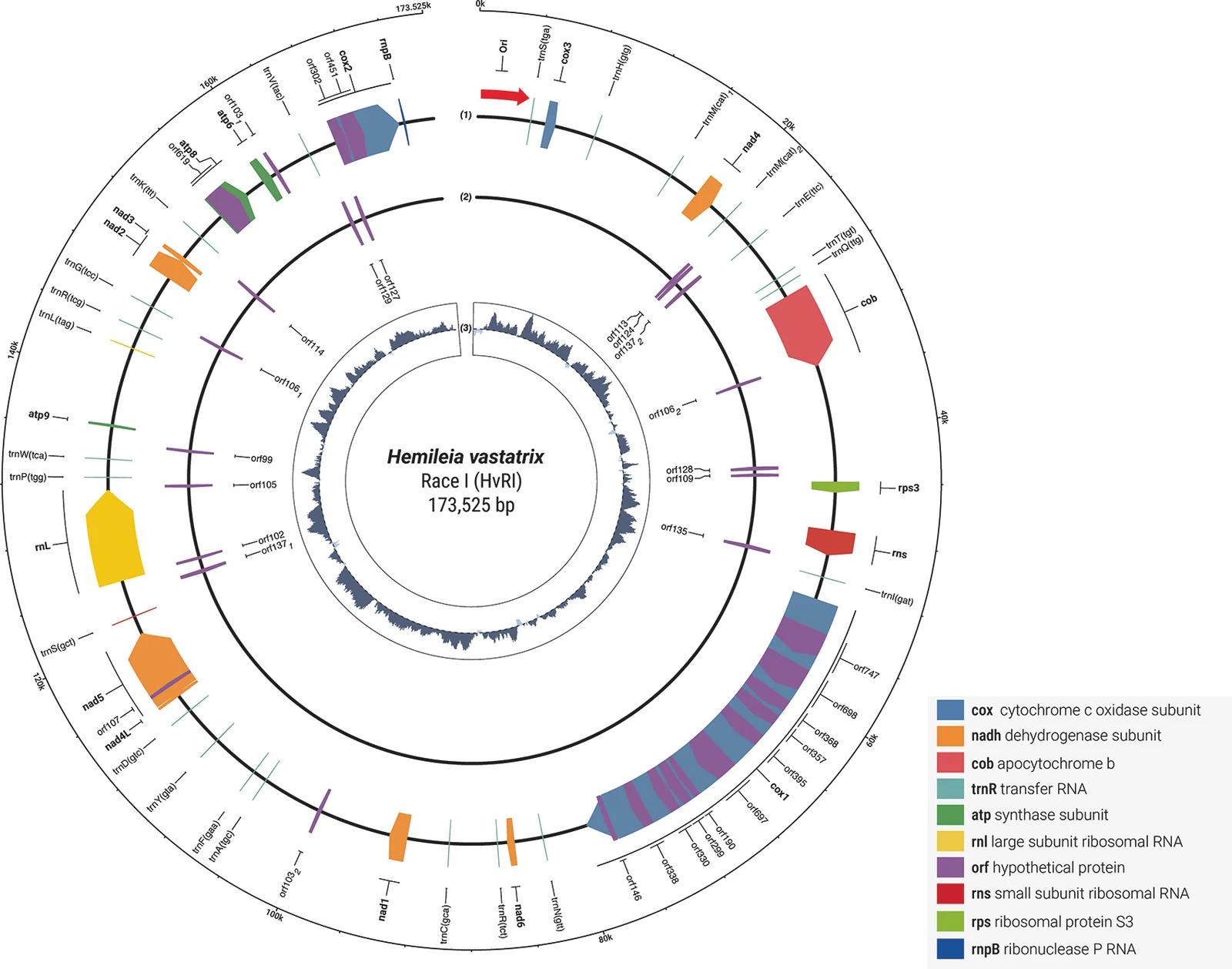
Circular map of the *Hemileia vastatrix* Race I mitochondrial genome (173,525 bp). Tracks are arranged from outermost to innermost: (1) Genes encoded on the forward (+) strand; (2) Genes encoded on the reverse (−) strand; and (3) GC skew calculated using a 400 bp sliding window (dark gray: positive values; light gray: negative values). Gene blocks are color-coded by functional category. The *rnpB* gene is located near the 170.6 kb mark on the outer track. Radial tick marks indicate genomic coordinates in kilobases (kb). The map is oriented such that the putative replication initiation zone (approx. 62 kb) is positioned at the top.

**Genome annotation and features**. The HvRI mitochondrial genome has a GC content of 33.1% typical of the order Pucciniales. Assembly metrics and genomic features are summarized in Table 1. Annotation identified a total of 41 functional genes, comprising 15 protein-coding genes (PCGs), two rRNAs ( *rnl* and *rns*), and 24 tRNAs ( Figure 1).

**Table 1.** Genome assembly metrics and annotation features of the Hemileia vastatrix Race I mitochondrial genome.

Property	Value
Organism	*Hemileia vastatrix Berk. and Br.*
Isolate	Race I (HvRI)
BioSample Accession	SAMN32232808
Genome Size	173,525 bp
Topology	Circular
GC Content	33.10%
Gene Content	41 functional genes (15 PCGs, 2 rRNAs, 24 tRNAs)
Sequencing Technology	PacBio Sequel II (HiFi); BGIseq/DNBseq (PE 150 bp)
Assembly Tools	NextDenovo v2.5.5; NOVOPlasty v4.3.5; NOVOLoci v0.5
Coverage Depth	1,240X (PacBio HiFi); 2,411X (BGIseq)
Raw Data Accession (SRA)	SRR22911636 (PacBio); SRR22911637 (BGIseq)
GenBank Accession	PX759424

The genome is characterized by significant structural complexity, particularly in the cytochrome *c* oxidase subunit I ( *cox1*) and *cob* genes, which contain multiple introns harboring LAGLIDADG and GIY-YIG homing endonucleases. The expansion of these intronic regions and intergenic spacers contributes to the large genome size observed in HvRI mitochondrial genome compared to those of other rust fungi, which ranges from 31,825 bp in *Phakopsora pachyrhizi* ^31^ to 102,521 bp of *Puccinia striiformis* f. sp. *tritici.* ^32^

Of particular interest is the ATP synthase subunit 8 ( *atp8*) gene. While introns are rare in fungal *atp8* genes, this locus contains an intron. BLASTX analysis of this region revealed sequence similarity to intron-encoded proteins, providing evidence of intron acquisition through group II intron retrohoming, likely mediated by a reverse transcriptase/maturase. Furthermore, the intronic ORF appears to have accumulated nonsense mutations resulting in several internal stop codons, indicating that this intronic element may be undergoing pseudogenization or functional loss in this isolate.

The putative replication initiation zone was identified within an approximately 1,140-bp region, beginning at position 62,683. The 5′ boundary is defined by a poly-G homopolymer, which serves as a potential initiation site for RNA priming. Downstream of this signal, we identified two copies of the conserved regulatory motif AGACCCGACC. The regulatory zone ends with the second copy of the conserved motif at position 63,779, serving as a replication checkpoint. The 3′ boundary is defined by a distinct AT-rich melting peak (maximum AT at position 63,817), after which the sequence composition reverts to standard GC content.

**Figure 2.**
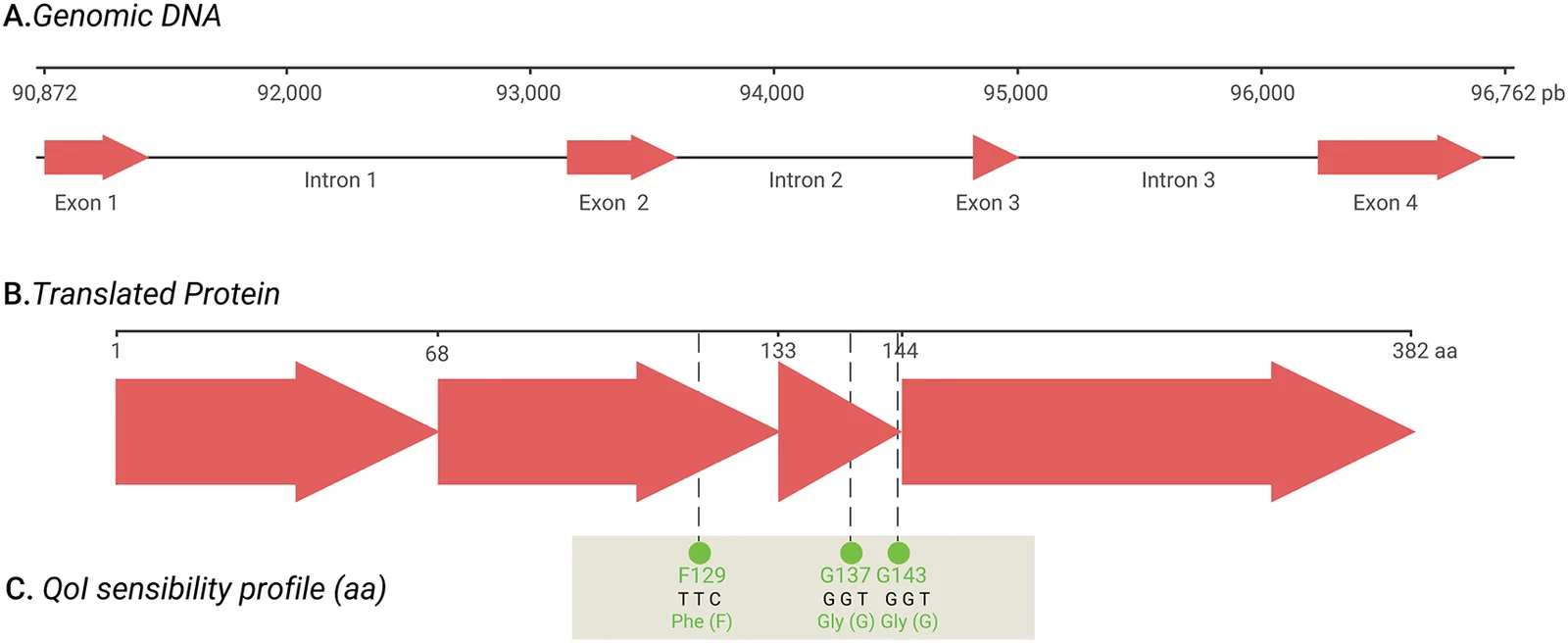
The *cob* gene in *Hemileia vastatrix* Race I. The schematic illustrates the transition from the genomic DNA scale (bp) to the functional protein scale (aa). A. The genomic track shows four exons separated by extensive introns, spanning coordinates 90,872 to 96,762 bp. B. In silico splicing yields a 382-residue protein. C. The quinone outside inhibitor (QoI) susceptibility profile identifies three critical wild-type markers: F129 (TTC), G137 (GGT), and G143 (GGT). No QoI resistance-associated substitutions were detected, establishing this assembly as a susceptible reference for coffee leaf rust.

Analysis of the *cob* gene architecture in the HvRI mitogenome revealed a conserved wild-type allelic configuration. Specifically, mutations that lead to non-synonymous substitutions associated with QoI fungicide resistance are absent in this isolate. The genomic coordinates for these markers were localized within Exon 2 (codon 129) and Exon 3 (codons 137 and 143), as illustrated in the structural mapping of the gene ( Figure 2). It will be important to characterize the *cob* sequence of isolates collected in coffee fields with reported fungicide control failures.

## Ethical approval

Ethical approval and consent were not required.

Large Language Models (Gemini Advanced/ChatGPT) were used exclusively for grammatical editing and language refinement of the author’s original text. The authors reviewed and take full responsibility for the content.

## Data Availability

The mitochondrial genome sequence was deposited in GenBank under the accession number PX759424.1. The associated Bio-Sample and SRA IDs are SAMN32232808, SRR22911637, and SRR22911637 respectively. NCBI GenBank: Hemileia vastatrix Race I mitochondrion, complete genome. Accession number PX759424. https://www.ncbi.nlm.nih.gov/nuccore/PX759424.1 (Marin-Ramirez; Maldonado; Padilla and Brommonschenkel, 2026). NCBI BioSample: MIGS Eukaryotic sample from *Hemileia vastatrix.* Accession number SAMN32232808. NCBI Sequence Read Archive (SRA): PacBio HiFi WGS of *Hemileia vastatrix* Race I: spores. Accession number SRR22911636. https://www.ncbi.nlm.nih.gov/sra/SRX18869071 NCBI Sequence Read Archive (SRA): BGIseq of *Hemileia vastatrix* Race I: spores. Accession number SRR22911637. https://www.ncbi.nlm.nih.gov/sra/SRX18869070
