# Supplementary material for: The complete and annotated mitochondrial genome of Hemileia vastatrix Race I, causal agent of coffee leaf rust
Source: F1000Res. 2026 Jul 15;15:759. Originally published 2026 May 19. [Version 2] doi: 10.12688/f1000research.177569.2 (PMC13550922; doi:10.12688/f1000research.177569.2)
Supplement: Peer review history

# Reviewer report: Reviewer response for version 2

**DOI:** 10.5256/f1000research.206040.r501799

**Published:** 2026-07-16

**Authors:** Theo Llewellyn

**Affiliations:** 1 Imperial College London Department of Life Sciences, London, England, UK; 2 Comparative Fungal Biology, Jodrell Laboratory Royal Botanic Gardens Kew Richmond, England, UK

No further comments.

Are the datasets clearly presented in a usable and accessible format, and the assembly and annotation available in an appropriate subject-specific repository?

Yes

Are sufficient details of the sequencing and extraction, software used, and materials provided to allow replication by others?

Partly

Are the rationale for sequencing the genome and the species significance clearly described?

Yes

Are the protocols appropriate and is the work technically sound?

Yes

Reviewer Expertise:

Fungal genomics, secondary metabolism, evolutionary ecology, fungal phylogenetics, lichenology, symbiosis

I confirm that I have read this submission and believe that I have an appropriate level of expertise to confirm that it is of an acceptable scientific standard.

# Reviewer report: Reviewer response for version 1

**DOI:** 10.5256/f1000research.195807.r494852

**Published:** 2026-07-07

**Authors:** Martin Coetzee

**Affiliations:** 1 University of Pretoria, Pretoria, South Africa

The manuscript is well written, and the mitochondrial genome reported here will be a valuable resource for future research on *Hemileia vastatrix* Berk. & Broome. In agreement with the other reviewer, I do not find any major flaws in the methods or results. I also have no major concerns about the manuscript and no substantial additions to the comments already provided by the previous reviewer. However, the authors should still address the remaining comments raised in the earlier review.

Are the datasets clearly presented in a usable and accessible format, and the assembly and annotation available in an appropriate subject-specific repository?

Yes

Are sufficient details of the sequencing and extraction, software used, and materials provided to allow replication by others?

Yes

Are the rationale for sequencing the genome and the species significance clearly described?

Yes

Are the protocols appropriate and is the work technically sound?

Yes

Reviewer Expertise:

Microbial genomics and evolution

I confirm that I have read this submission and believe that I have an appropriate level of expertise to confirm that it is of an acceptable scientific standard.

# Response

**Published:** 2026-07-08

**Authors:** Gustavo Marin

**Affiliations:** Plant Pathology, Federal University of Vicosa, Viçosa, State of Minas Gerais, Brazil

Dear Prof. **Martin Coetzee**,

 We sincerely thank the reviewer for the positive evaluation of our manuscript and for recognizing the value of the mitochondrial genome presented here for future research on *Hemileia vastatrix*. We are also grateful for the reviewer’s agreement that there are no major concerns regarding the methods or results.

 As suggested, we have carefully addressed all remaining comments raised by **Reviewer **(Theo Llewellyn) in the revised manuscript, including clarification of the wording regarding fungal mitochondrial evolutionary rates and the addition of the quality-filtering and polishing details in the Methods section. These revisions have improved the precision, reproducibility, and overall clarity of the manuscript

 Sincerely,  

 On behalf of all authors

# Reviewer report: Reviewer response for version 1

**DOI:** 10.5256/f1000research.195807.r489837

**Published:** 2026-06-10

**Authors:** Theo Llewellyn

**Affiliations:** 1 Imperial College London Department of Life Sciences, London, England, UK; 2 Comparative Fungal Biology, Jodrell Laboratory Royal Botanic Gardens Kew Richmond, England, UK

The authors provide a concise and well-written article detailing a new high-quality mitochondrial genome sequence for the causal agent of coffee leaf rust *Hemileia vastatrix*. There are no major flaws I could identify and only a couple of minor points to address, which I have listed below.

 Paragraph 2: 'the opposite is often observed in fungi' this sentence requires a citation. It also feels overly generalised as mitochondrial evolutionary rates vary across the kingdom. Whilst this may be true for some asexual *Basidiomycota*, I think 'often observed in fungi' does not accurately represent this.

 Methods: which quality-filtering thresholds and tools were applied to reads that were then mapped back to contigs with pilon? I could not find this in the text

 Figure 1: needs a colour legend for the functional categories

Are the datasets clearly presented in a usable and accessible format, and the assembly and annotation available in an appropriate subject-specific repository?

Yes

Are sufficient details of the sequencing and extraction, software used, and materials provided to allow replication by others?

Partly

Are the rationale for sequencing the genome and the species significance clearly described?

Yes

Are the protocols appropriate and is the work technically sound?

Yes

Reviewer Expertise:

Fungal genomics, secondary metabolism, evolutionary ecology, fungal phylogenetics, lichenology, symbiosis

I confirm that I have read this submission and believe that I have an appropriate level of expertise to confirm that it is of an acceptable scientific standard.

# Response

**Published:** 2026-07-08

**Authors:** Gustavo Marin

**Affiliations:** Plant Pathology, Federal University of Vicosa, Viçosa, State of Minas Gerais, Brazil

Dear Prof. **Theo Llewellyn**,

 We would like to sincerely thank you for the careful evaluation of our manuscript and for the constructive comments and suggestions. We greatly appreciate the time and effort invested in reviewing our work. Your feedback has helped us improve the clarity, precision, and methodological transparency of the manuscript. Below, we provide a point-by-point response to the comments.

**Comment 1**

 “the opposite is often observed in fungi” this sentence requires a citation. It also feels overly generalised as mitochondrial evolutionary rates vary across the kingdom. Whilst this may be true for some asexual Basidiomycota, I think ‘often observed in fungi’ does not accurately represent this.

- **Response**: We agree that our original wording was too broad and could be interpreted as overgeneralizing mitochondrial evolutionary patterns across fungi. In response, we revised the sentence to be more precise and cautious, and we added appropriate citations supporting the observation that, in some fungal lineages, nuclear genomes may exhibit higher polymorphism than mitochondrial genomes, while acknowledging that these patterns are not universal across the kingdom. *We replaced the original statement with a more nuanced phrasing that avoids generalization and reflects the heterogeneity of mitochondrial evolutionary rates in fungi.*

**Comment 2**

 Methods: which quality-filtering thresholds and tools were applied to reads that were then mapped back to contigs with pilon? I could not find this in the text

- **Response:** We have revised the Methods section to explicitly describe the preprocessing and polishing workflow. Specifically, the raw BGIseq reads were adapter-trimmed and converted to Phred+33 format using Trimmomatic v0.40. After the initial assembly with NOVOPlasty v4.3.5, the reads were further quality-trimmed prior to polishing using Trimmomatic with the following parameters: leading and trailing base quality < 20, a 4-bp sliding window requiring an average quality score of at least 30, and a minimum read length of 100 bp. The filtered reads were then aligned to the draft assembly with BWA-MEM v0.7.17 and the assembly was polished with Pilon v1.24. *To improve clarity, we also suggest incorporating the following text into the manuscript.*

We again thank the reviewer for the constructive feedback, which has significantly improved the manuscript. We hope that the revisions adequately address the concerns raised and enhance the quality and reproducibility of our study.

 Sincerely,  

 On behalf of all authors.

# Response

**Published:** 2026-07-09

**Authors:** Gustavo Marin

**Affiliations:** Plant Pathology, Federal University of Vicosa, Viçosa, State of Minas Gerais, Brazil

Dear **Reviewers**,

 Paragraph 2: 'the opposite is often observed in fungi' this sentence requires a citation. It also feels overly generalised as mitochondrial evolutionary rates vary across the kingdom. Whilst this may be true for some asexual Basidiomycota, I think 'often observed in fungi' does not accurately represent this.

 Mitochondria are double-membrane organelles, present in high copy numbers per cell. They are essential for fungal metabolism and the site for oxidative phosphorylation.3 In sexual eukaryotes, mitochondrial inheritance is predominantly uniparental; however, in fungi, both uniparental and biparental inheritance modes have been reported.4 Another distinction across the tree of life is the mutation rate: while mitochondrial genomes evolve faster than nuclear genomes in animals, the opposite is often observed in fungi. Predominantly asexual species of the division Basidiomycota present SNP frequencies up to 7.69% in nuclear genomes but only up to 4.41% in their mitochondrial counterpar

 ts.5 This slower mutation rate makes mitochondrial DNA markers useful for resolving deep evolutionary relationships between species.6–8 Despite the slow mutation rate in Basidiomycota, high variability in genome size, gene order, and intron content has been found while maintaining a conserved core set of protein-coding genes, this genomic plasticity is valuable not only for phylogenetics of distant species but also for population genomics within a species.9

**Response**

 We agree with the reviewer that our original statement was overly generalized, given the vast evolutionary diversity within the fungal kingdom. We have revised the text to clarify that slower mitochondrial evolution is not universal but has been documented in specific lineages, seamlessly leading into our cited example of Basidiomycota (Reference 5).

 Reviewer comment:

 Methods: which quality-filtering thresholds and tools were applied to reads that were then mapped back to contigs with pilon? I could not find this in the text

 Original paragraph:

 The mitochondrial genome was assembled using a hybrid approach integrating multiple tools. Initially, a de novo assembly was performed using NOVOPlasty v4.3.516 with BGIseq reads (SRA SRR22911637), using the cob gene sequence of Puccinia striiformis f. sp. tritici isolate CYR32 (GenBank MH891489.1) as a seed. The resulting contig was annotated with MFannot v2.017 using Genetic Code 4 (Mold, Protozoan, and Coelenterate Mitochondrial). The NADH dehydrogenase subunit 4 (nad4) gene from this preliminary assembly was then used as a seed for a second round of NOVOPlasty. Finally, the assembly was polished with Pilon v1.2418 by mapping quality-trimmed reads back to the draft assembly using BWA-MEM v0.7.17.19

 Edited paragraph:

 The mitochondrial genome was assembled using a hybrid approach. Initially, raw BGIseq reads (SRA SRR22911637) were adapter-trimmed using Trimmomatic v0.40 (Beier et al., 2026). A de novo assembly was then performed using NOVOPlasty v4.3.516, which utilizes internal read quality filtering, using the cob gene sequence of Puccinia striiformis f. sp. tritici isolate CYR32 (GenBank MH891489.1) as a seed. The resulting contig was annotated with MFannot v2.017 using Genetic Code 4 (Mold, Protozoan, and Coelenterate Mitochondrial). The NADH dehydrogenase subunit 4 (nad4) gene from this preliminary assembly was subsequently used as a seed for a second round of NOVOPlasty. Finally, prior to error correction, the raw reads underwent quality trimming with Trimmomatic (leading/trailing Q < 20; 4-bp sliding window average Q ≥ 30; minimum length 100 bp). These high-quality reads were mapped to the draft assembly using BWA-MEM v0.7.17, and the assembly was polished with Pilon v1.24.

 Beier, S., Bolger, A.M., Bolger, M.E., Schwacke, R. and Usadel, B., 2026. Trimmomatic: a decade of feature-rich, high-performance NGS read preprocessing. Bioinformatics, 42(6), p.btag331.

 Reviewer Comment:

 "Methods: which quality-filtering thresholds and tools were applied to reads that were then mapped back to contigs with pilon? I could not find this in the text"

**Response:**

 We thank the reviewer for pointing this out. This comment made us realize that the initial data preprocessing was not clearly explained. We have updated the Methods section to clarify how the paired-end data was handled in two stages. First, we removed adapters using Trimmomatic v0.40, allowing NOVOplasty to use its own internal read filtering for the assembly. Second, we clarified that a strict quality filtering step (leading/trailing Q < 20; 4-bp sliding window average Q ≥ 30; minimum length 100 bp) was applied using Trimmomatic prior to mapping the reads for Pilon polishing.
